# Update on House Dust Mite Allergen Avoidance Measures for Asthma

**DOI:** 10.1007/s11882-020-00948-y

**Published:** 2020-06-19

**Authors:** Chiara Zuiani, Adnan Custovic

**Affiliations:** 1 Imperial College Healthcare NHS Trust, London, UK; 2 National Heart and Lung Institute, Imperial College London, London, UK

**Keywords:** House dust mite, Asthma, Avoidance, Exposure, Allergen, Mite

## Abstract

**Purpose of Review:**

To critically review the evidence in favor or against the use of house dust mite (HDM) allergen avoidance measures in patients with asthma.

**Recent Findings:**

Systematic reviews and meta-analyses suggested no positive effect of mite allergen avoidance strategies on asthma outcomes, resulting in a lack of consensus regarding the utility of these measures. However, such analyses have a number limitations and might not be the most adequate tool to evaluate current evidence and to derive clinical recommendations regarding mite allergen avoidance in asthmatic patients. We should not disproportionately rely on the results of meta-analyses and systematic reviews to inform clinical practice and asthma guidelines in this area. Recent high-quality evidence from randomized controlled trial in children confirmed that mite allergen–impermeable bed encasings reduce emergency hospital attendance with acute severe asthma exacerbations.

**Summary:**

Until better evidence is available, we suggest that physicians should adopt a pragmatic approach to mite allergen avoidance and advise sensitized patients to implement a multifaceted set of measures to achieve as great a reduction in exposure as possible. Potential predictors of positive response (e.g., patient’s sensitization and exposure status) can pragmatically be evaluated using the size of skin test wheal or the titer of allergen-specific IgE. Finally, the intervention should be started as early as possible.

## Introduction

Sensitization to house dust mite (HDM) is one of the most common causes of respiratory allergy in the world [1] and has been consistently found to be one of the strongest associates of asthma in children, adolescent, and adults [2–5]. Asthma symptoms in children and adults sensitized to HDM tend to correlate with mite allergen levels at home [6, 7]. High domestic exposure to mite allergens in asthmatic patients with HDM sensitization triggers bronchospasm and increases bronchial hyper-reactivity, whereas cessation of exposure may relieve symptoms [8]. Therefore, it appears intuitive that avoidance should be recommended as part of the clinical management of HDM-sensitized patients with allergic asthma and rhinitis. Different methods for reducing mite exposure have been evaluated, such as physical barriers, chemical methods, or the combinations of both. However, most studies evaluating the impact of single avoidance interventions or multicomponent strategies have failed to show improvements in main asthma outcomes. A number of systematic reviews and meta-analyses have questioned the clinical utility of measures designed to reduce HDM exposure in asthmatic patient, resulting in a lack of consensus and conflicting recommendations by the national and international asthma guidelines. As an example, the US NHLBI Asthma Guideline (2007) provides the opposite recommendations on the use of HDM avoidance in the management of the disease compared to the Global Initiative for Asthma (GINA), the British guideline on the management of asthma (BTS/SIGN), or the UK National Institute of Clinical Excellence (NICE) guideline on asthma [9–12]. The most recent guidelines recommendations on the use of HDM avoidance in asthma are summarized in Table 1.

**Table 1:** Recommendations regarding allergen avoidance according to different asthma guidelines

Guideline	Advice regarding allergen avoidance
NHLBI (2007) [9]	“Recommend multifaceted approaches to control exposures to which the patient is sensitive; single steps alone are generally ineffective.”
GINA (2020) [10]	“Allergen avoidance is not recommended as a general strategy in asthma. For sensitized patients, there is limited evidence of clinical benefit for asthma in most circumstances with single-strategy indoor allergen avoidance.”
BTS/SIGN (2019) [11]	“Physical and chemical methods of reducing house dust mite levels in the home (including acaricides, mattress covers, vacuum cleaning, heating, ventilation, freezing, washing, air filtration, and ionizers) should not be routinely recommended by healthcare professionals for the management of asthma.”
NICE (2017) [12]	Emphasis on pharmacological treatment; no mention of environmental control measures.

We have recently reviewed this topic, outlining the potential reasons for such discrepancies [13]. In this article, we provide an update on this subject, review the recent evidence which may impact upon recommendations in future iteration of guidelines, suggest a simple approach to mite allergen avoidance for practicing physicians, and discuss the potential ways to identify patients who may benefit from reduction in personal exposure to HDM.

## House Dust Mite Exposure, Sensitization, and Asthma

The relationship between exposure to dust mites and the development of specific sensitization and asthma is complex, with previous studies reporting inconsistent findings (reviewed in detail in [14]). Two different cohort studies showed an apparent dose-response relationship between high HDM exposure in infancy and development of asthma or wheezing at school age [15, 16], while a third one detected this only in a subgroup of children with a family history of allergic disease [17]. The findings from the “Childhood Asthma Prevention Study” [18] further suggested a non-linear “bell-shaped” relationship between HDM exposure and asthma, with very high or very low level of HDM exposure in the household being both protective against asthma. On the other hand, several other birth cohort studies failed to demonstrate any relationship between early allergen exposure and subsequent development of asthma [19–21].

There are many possible reasons for such divergent findings. First, available studies are difficult to compare due to major differences in study design, outcome measures, and methods of assessing exposure. Second, differences in geographic location and patterns of HDM allergen exposure and relevant co-exposures could have affected reported relationships. Furthermore, genetic background of the study populations impacting upon susceptibility to HDM allergen exposure may also differ between studies, potentially contributing to poor repeatability of results. Finally, from current evidence, it is unclear whether the time in life when individuals are exposed to high HDM levels may affect the risk of future asthma and, consequently, at what age allergen exposure should be preferentially assessed.

The route of exposure to HDM allergens is considered to be primarily inhaled. However, especially in children, other routes might have a role. The oral route could be relevant for young children who frequently put hands and toys to the mouth and may ingest more than 100 mg/day of dust [22, 23]. Sensitization may also occur through an impaired skin barrier. This may be of particular importance within the context of *filaggrin* loss-of-function mutations, as it was shown for environmental exposure and sensitization to peanut allergen [24]. Similarly, for HDM allergy, recent analysis in a population-based birth cohort has shown that among children exposed to mite allergen, those with *FLG* mutations had a much higher risk of being sensitized to HDM by 1 year of age (OR 6.66, 95% CI 1.15–38.58) [25••].

Another issue complicating the interpretation of current data on the relationship between HDM exposure and asthma risk is the absence of a reliable indicator of personal allergens exposure. In most studies, the personal exposure is assumed to equate to the concentration of allergen measured in dust samples collected by vacuuming a single square meter of settled dust on carpet or patient’s mattress. This assumption is questionable, particularly in light of evidence showing that HDM allergen levels may vary considerably within the same house and within the same sampling site (e.g., mite allergen in different parts of the same carpet may vary as much as 100-fold) [26]. Therefore, a standardized and reliable measure which can accurately reflect personal HDM exposure is urgently needed [27]. In fact, in the authors’ opinion, only an accurate marker of personal exposure investigated in the specific context of patients’ genetic and biological background will provide a better understanding of the role of allergens in the development of allergic disease.

The evidence that high exposure to mite allergens adversely impact asthma severity in at least some mite-allergic asthmatics is much more consistent [7, 28–30]. High allergen exposure interacts with virus infections to increase the risk of hospital admissions for asthma in children [31] and adults [32]. There is a limited but intriguing evidence that high mite exposure increases asthma severity among non-atopic asthmatics [33]. The evidence of the negative impact of high mite exposure on asthma control provides rationale for the use of mite avoidance in asthma management.

## House Dust Mite Avoidance Strategies

Dust mite allergens can be detected in most dust reservoirs, including beds, carpets, sofas, drapes, soft toys, and clothing [34]. The main allergens are immune-stimulatory proteins that are contained in dust mite feces [35, 36]. Mite allergens are mostly carried on large particles (diameter > 10 μm) and are usually not detectable in undisturbed conditions in ambient air [37]. However, disturbance of the dust reservoirs, such as vacuum cleaning or bed-making, can aerosolize dust for as long as 15 min and increase the amount of inhaled allergen [38].

A myriad of dust mite avoidance measures have been proposed, including mattress and pillow encasings, high-efficiency particulate air filtration (HEPA) vacuum cleaners, air purification, acaricides, humidity control, and physical removal of mite reservoirs [39••]. The most common strategies that have been used to control domestic allergen exposure are physical barriers such as covers for pillows, duvet, and mattress. Covers made of woven fabrics with a pore size up to 6 μm are very effective at controlling mite allergens passage [40]. The use of dust mite-impermeable bedding covers as an isolated intervention has been repeatedly shown to be an effective measure in reducing the amount of dust mite allergen recovered from the bed/bedding surface [41–43]. HDM-impermeable covers for mattress, duvets, and pillow are one of the few mite avoidance measures which have been subjected to a rigorous double-blind placebo-controlled trials to assess their clinical efficacy on various asthma outcomes. For example, a randomized double-blind, placebo-controlled trial has shown a reduction of mite allergen load by 98.5% and 95.4% in the active group after 1 and 9 weeks of intervention, which was mirrored by an improvement in the morning peak flow [43].

In general, feather pillows are preferable to those made of synthetic materials (likely because the covers of feather pillows are woven much tightly compared to the synthetic ones) [44]. All bedding should be washed frequently (e.g., on a weekly basis in a hot cycle above 55 °C to kill dust mites and eliminate the residual allergens) [45]. Ideally, the presence of carpets, upholstery, and stuffed toys should be minimized in the houses of patients allergic to HDM in order to reduce the reservoir for mite colonization. Regular vacuuming of floors using a vacuum equipped with HEPA filter is often recommended, although patients should avoid the process of emptying the dust compartments of the vacuum cleaner as this causes a burst of high mite exposure [46].

The use of acaricides has been investigated more than 3 decades ago, but it only led to a modest decrease in mite allergen concentration when applied on carpets [47]. Air filtration does not seem to have a measurable impact on reducing mite exposure or improving symptoms in mite sensitized patient with perennial allergic rhinitis and asthma, presumably because mite allergens are seldom airborne [48, 49].

Reducing humidity can decrease dust mite growth, but a target of relative humidity consistently well below 50% should be pursued for this intervention to be effective [50]. The success of methods to achieve this varies according to the design of houses and climate [51]. Portable dehumidifiers did not show any effect on dust mite presence or mite allergen level in temperate climate with high relative humidity [52].

The identification of sources of mite exposure is important to design effective avoidance strategies. Most mite avoidance strategies were developed under the assumption that HDM exposure among individual patients mainly occurs during time spent in bed, on the floor, or on upholstered furniture. However, a study by Tovey et al. challenged this belief, showing that most of individual mite allergen exposure in group of adults living in an urban environment in Australia occurred while commuting on public transport [53]. It is very likely that the actual individual exposure is different in adults compared to children and, consequently, different age groups may have differential response to avoidance measures. For example, it is possible that mattress and carpets are the main mite reservoirs and source of exposure for younger children who spend most of the time at home, spend more time in bed or on the floor than adults, whereas other sources of HDM may play an important role in adults [53]. Similar to the unfounded assumptions on how and when exposure to dust mite occurs and the effectiveness of measures to reduce personal exposure, unfounded inference is often made about the clinical effectiveness of house dust mite allergen avoidance measures.

## Clinical Effectiveness of House Dust Mite Allergen Avoidance Measures

### Systematic Reviews and Meta-Analyses

Several systematic reviews and meta-analyses concluded that the body of available evidence failed to demonstrate the effectiveness of mite allergen avoidance to improve asthma outcomes [54–57]. These publications had a considerable impact on the recommendation by various asthma guidelines, which overly rely on the results of meta-analyses and systematic reviews to inform clinical practice. The most recent Cochrane meta-analysis published in 2008 included 54 clinical trials which evaluated the efficacy of chemical methods, physical barriers, or the combination of different mite allergen avoidance measures on asthma control; the authors concluded that mite avoidance had no significant benefit on different asthma outcomes (lung function, symptoms, or anti-asthma medications use) [56]. The authors concluded that strategies aimed to reduce exposure to mite allergens cannot be recommended to mite-allergic patients with asthma, raising uncertainty and a considerable disagreement among clinicians and experts [58–61]. Furthermore, findings from a recent systematic reviews which included 67 trials (8 of which not randomized) evaluating single (37 trials) or multicomponent avoidance strategies (30 trials) showed inconclusive results or no benefit in asthma [57]. A Cochrane review of HDM avoidance measures for rhinitis reported that the evidence was insufficient to offer a definitive recommendation, as most published trials have been small and of questionable quality [62].

An important question for practicing physicians and the members of guideline committees is whether meta-analysis is the most appropriate tool to evaluate evidence about the efficacy of dust mite allergen avoidance. There is a number of potential problems and issues with published systematic reviews and meta-analyses regarding HDM avoidance in asthmatic patients, which have been highlighted in a thoughtful and insightful article by Tom Platts-Mills [61]. Limitations include the fact that results from studies of adults and children were combined in meta-analyses (a practice that is applied to any other intervention). For example, most physicians would agree that it would be fundamentally flawed to lump together studies of inhaled corticosteroids (ICS) in pre-school children with those in adult patients). We wish to emphasize that data for adults and children should be assessed separately, rather than together. The similar applies to different outcome measures, and the impact of intervention on different asthma domains (such as symptoms, exacerbations, lung function, quality of life, airway hyperreactivity) should not be reported together. Furthermore, studies with short intervention periods which are highly unlikely to have a realistic chance of showing a clinical effect were analyzed together with much longer trials (it is of note that studies of allergen avoidance at high altitude suggest that clinical benefits are apparent after several months). To use the same analogy of ICS, inclusion of studies lasting a week or two in joint analyses with long-term trials would not be considered a best practice. Studies evaluating multifaceted avoidance strategies were excluded from the analysis (reviewed in [13]). Several studies included in meta-analyses used methods which are ineffective in reducing mite exposure. Moreover, a recent meta-analysis evaluating baseline characteristics of patients who were included in mite avoidance trials showed a variable and sometimes negligible levels of mite allergen exposure at baseline [63••]. Reducing exposure in a patient not exposed in the first place is hardly going to be of benefit. A threshold of 10 μg/g dust has been suggested as being relevant to asthma symptoms [64] meaning that, on average, the baseline allergen level in many mite avoidance trials was low, even trivial in the same studies. Another potential problem was that a (considerable) proportion of participants had mild asthma, with little scope for improvement [63••]. The inclusion of trials of patients with mild and well-controlled asthma could conceal positive results, unless sub-analyses according to asthma severity are performed. Again, this contrasts the efficacy studies in ICS, most of which are conducted in carefully selected patients with suboptimal asthma control and other features that favor beneficial response (e.g., exclusion of smokers and those without reversibility to bronchodilators).

Given these and other issues (e.g., difficulties in blinding and maintaining interventions without extensive education for mite avoidance studies), it is crucially important to recognize the many limitations of meta-analyses and move away from over-reliance on meta-analyses and systematic reviews to inform clinical practice in this area of research and practice.

### Randomized Clinical Trials: Most Relevant Data

Given the potential problems with meta-analyses, it is worth discussing in more detail several randomized double-blind, placebo-controlled studies which tested interventions aimed at reducing mite allergen exposure. The largest trial to date which assessed the effectiveness of bed covers as a single intervention recruited more than 1000 adults with asthma (two thirds of whom were mite sensitized) found no benefits in either primary or secondary outcome measures (morning PEFR during the first 6 months, the proportion of patients able to discontinue ICS during months 7–12, symptoms scores, and quality of life) [65]. Similarly, the largest RCT of bed encasings in mite-sensitized adults with allergic rhinitis demonstrated no beneficial effect [66]. These two studies are often used as a proof that bed covers are ineffective in asthma management. However, failure to demonstrate benefit in some age groups or asthma domains (e.g., lung function or symptoms) does not exclude the benefit in other age groups and/or other domains (such as exacerbations). For example, in contrast to many studies in adults, most trials in children showed a positive impact of mite avoidance strategies [67••, 68–72]. Main findings of trials evaluating HDM avoiding strategies in HDM-sensitized children with asthmatic with are reported in Table 2.

**Table 2:** Selected evidence about house dust mite avoidance in pediatric asthma

Evidence	Sample size	Measures	Length of intervention	Findings
Murray et al [67••]	284	Physical barriers	1 year	Significant reduction of asthma exacerbation and risk of hospital visits
Morgan et al [68]	937	Multi-faced approach targeting multiple allergens (including physical barriers, vacuum cleaner with HEPA filter)	2 years	Significant reduction in asthma symptoms and A&E visits
Halken et al. [69]	60	Physical barriers	1 year	Reduction by at least 50% dose of inhaled steroids
Carter et al. [70]	104	Multi-faced approach targeting multiple allergens (including physical barriers, education)	1 year	Significant reduction in acute healthcare visits for asthma
Shapiro et al. [71]	36	Multi-faced approach (including acaricide, physical barriers, laundry service, education)	1 year	Improvement in bronchial hyper-responsiveness No changes in FEV1, symptom score and quality of life
Carswell et al. [72]	70	Multi-faced approach (including acaricide and physical barriers)	6 months	Improvement in FEV1, reduction in asthma medication and symptoms

The choice of asthma domain used as a primary outcome measure is also important. This has been highlighted in studies of biologicals in asthma; for example, mepolizumab had no effect on late-phase allergic reaction (and was deemed a failure) but was subsequently shown to substantially reduce severe exacerbations. Similarly, omalizumab has much bigger effect on asthma exacerbation rate than on symptom control or lung function.

A very important recent RCT in children (preventing asthma exacerbations by avoiding mite allergen—PAXAMA) tested the hypothesis that reduction in mite allergen exposure may reduce the risk of severe asthma exacerbations. The study enrolled 284 mite sensitized asthmatic children after being admitted to hospital with severe asthma exacerbation and randomized them to receive mite-impermeable bedding covers (*n* = 146) or placebo covers (*n* = 138). There was a significant reduction of severe asthma exacerbation during the 12-month follow-up in the active group, with 29% of children in the intervention group having an hospital visit for asthma compared with 42% of the control group. The intervention did not influence the use of oral corticosteroids [67••]. In a subgroup analysis, the greater benefit was observed in children younger than 11 years (*p* = 0.006), those not exposed to tobacco smoking (*p* = 0.02), and children who had more severe asthma (*p* = 0.03) and were mono-sensitized to dust mite (*p* = 0.04) [67••].

An American study on asthmatic children evaluated the efficacy of a combining different environmental control strategies tailored to the child’s allergen sensitization and exposure status [68]. The interventions included physical barriers, HEPA vacuum cleaner, education, and advice on the reduction of passive smoke exposure and further products. This approach resulted in a significant reduction in asthma symptoms and A&E visits in the intervention group, supporting the assumption that reducing allergen exposure in children with allergic asthma to HDM will improve symptoms.

Overall, the available evidence suggests that environmental control measures have been effective when they were tailored to the patient’s characteristics and home exposures and were effective in some patients, especially in children.

## Conclusions

Until better evidence is available, we suggest that practicing physicians should adopt a pragmatic approach to mite allergen avoidance and advise sensitized patients to implement a multifaceted set of measures and attempt to achieve as great a reduction in exposure as possible. If possible, the intervention should be individualized, and potential predictors of positive response (e.g., patient’s sensitization and exposure status) can pragmatically be evaluated using the size of skin test wheal or the titer of allergen-specific IgE. Finally, we advise that the intervention should be started as early as possible.
